# Component rotational mismatch in the standing position is a potential risk factor for unfavourable functional outcomes after total knee arthroplasty

**DOI:** 10.1002/jeo2.12069

**Published:** 2024-07-02

**Authors:** Yasuhiko Kokubu, Shinya Kawahara, Hideki Mizu‐Uchi, Satoshi Hamai, Yukio Akasaki, Taishi Sato, Shojiro Ishibashi, Toshiki Konishi, Yasuharu Nakashima

**Affiliations:** ^1^ Department of Orthopaedic Surgery, Graduate School of Medical Sciences Kyushu University Fukuoka Japan; ^2^ Department of Orthopaedic Surgery Saiseikai Fukuoka General Hospital Fukuoka Japan

**Keywords:** rotational mismatch, standing position, total knee arthroplasty

## Abstract

**Purpose:**

This study assessed rotational mismatch between components after total knee arthroplasty (TKA) in the supine and standing positions and aimed to investigate the effect of rotational mismatch in the standing position on postoperative patient‐reported outcome measures (PROMs).

**Methods:**

Seventy‐one patients (71 knees) who underwent TKA for medial knee osteoarthritis were used to investigate rotational mismatches between components. Rotational mismatches between components were examined on postoperative standing whole‐leg and supine knee radiographs using a three‐dimensional‐to‐two‐dimensional model image registration technique, and the angles between the reference axes of the components were measured. Component alignment was evaluated using postoperative computed tomography images, and a questionnaire (2011 version of the Knee Society Score: [KSS 2011]) was mailed to investigate postoperative PROMs.

**Results:**

In the entire cohort, rotational mismatches in the supine and standing positions were similar (*p* = 0.9315). In 15% of patients, the mismatch was large (>5°) in the supine position but small (<5°) in the standing position (overestimated group). However, in 23% of patients, the mismatch was small (<5°) in the supine position and large (>5°) in the standing position (underestimated group). The underestimated group had severe preoperative varus deformity, resulting in external rotation of both femoral and tibial components. Rotational mismatch in the standing position (*p* = 0.0032) was a significant risk factor for unfavourable PROMs. Patients with a mismatch in the standing position had significantly lower scores than those without a mismatch (*p* = 0.0215), exceeding the minimal clinically important difference values.

**Conclusions:**

The underestimated group is clinically important because the surgical procedure and intraoperative assessment of component placement are performed in the supine position. In cases of severe preoperative varus deformity, care should be taken not to place the component in malrotation to avoid rotational mismatch in the standing position.

**Level of Evidence:**

Ⅳ, Case series.

**Abbreviations**
2D: two‐dimensional
3D: three‐dimensional
ACL: anterior cruciate ligament
AP: anteroposterior
CAD: computer‐aided design
CT: computed tomography
DICOM: Digital Imaging and Communications in Medicine
HKA: hip‐knee‐ankle
OA: osteoarthritis
PROM: patient‐reported outcome measure
SEA: surgical epicondyle axis
TKA: total knee arthroplasty

## INTRODUCTION

Total knee arthroplasty (TKA) is an effective procedure for treating end‐stage knee osteoarthritis (OA). However, postoperative rotational malalignment of this component can lead to maltracking [2], instability [44], anterior knee pain [3] and poor functional outcomes [14]. Although there have been systematic reviews on rotational alignment following TKA [30, 44], there is no clear definition of reference values for the rotational mismatch between the femoral and tibial components. Some studies suggest that a rotation mismatch of >5° between the components should be avoided after TKA due to concerns about anterior knee pain [1, 3] and biomechanical factors related to controlling external rotation of the femur during knee flexion [1, 10].

Additionally, all previous studies evaluated the rotational alignment by analysing computed tomography (CT) images in the supine position [30, 44]. However, evaluating the alignment in the standing position is clinically important because it represents a weight‐bearing functional limb position. Rotational alignment between components may differ between standing and supine evaluations. It has been expected that there would be a smaller rotational mismatch in the weight‐bearing position due to increased constraints between the components [43]. However, no reports have compared these two positions. It is clinically relevant to investigate the cases where there is a significant change in rotational alignment between the supine and standing positions because intraoperative component placement and preoperative/postoperative imaging evaluations are primarily performed in the supine position.

This study aimed to (1) assess the rotational mismatch between components in the supine and standing positions and (2) investigate the effect of rotational mismatch in the standing position on postoperative patient‐reported outcome measures (PROMs). The hypotheses were as follows: rotational mismatch differs between the supine and standing positions in some cases, and a rotational mismatch of >5° between components in the standing position would be a risk factor for unfavourable postoperative PROMs through maltracking, instability and anterior knee pain.

## MATERIALS AND METHODS

### Ethics statements

The local institutional review board approved the study procedures (number: 2020‐204) and conducted them according to the 1964 Declaration of Helsinki. All included patients provided informed consent.

### Patients

This is a case series with a level of evidence of 4. We retrospectively analysed consecutive patients who underwent TKA between April 2014 and December 2019. Inclusion criteria comprised patients experiencing disabling knee pain and diagnosed with medial knee OA of Kellgren–Lawrence Grade 3 or 4. Exclusion criteria included: (1) bilateral TKA recipients; (2) individuals with a history of high tibial osteotomy; (3) those with neuromuscular disease; (4) non‐availability of postoperative PROMs; (5) occurrence of postoperative complications and (6) patients with symptomatic contralateral knee OA or ipsilateral hip OA. Postoperative PROMs were gathered via mail using the 2011 version of the Knee Society Score (KSS 2011), a widely utilized assessment tool [33, 39]. Knee extension and flexion angles were measured using a two‐arm goniometer. The assessments were conducted in the supine position both preoperatively and at the latest postoperative follow‐up visit. The latest postoperative follow‐up visits all took place within 4 months of the questionnaire mailing.

### Surgical technique

All patients underwent TKA with a cemented posterior‐stabilized design (Persona; Zimmer Biomet) using the same standardized technique. The surgery was performed by a team of two experienced surgeons (SK and HM) using a measured resection technique with mechanical alignment. The components were aligned based on the preoperative planning on CT images using ZedKnee software (Lexi Co., Ltd.). The components were aligned perpendicular to the mechanical axis in the coronal plane. The femoral component was aligned perpendicular to the anatomical axis in the sagittal plane and parallel to the surgical epicondyle axis (SEA) in the axial plane. The tibial component was placed in the sagittal plane with a 3° posterior tilt from the anatomical axis. The rotational alignment of the tibial component was parallel to ‘Akagi's line’, which is the anatomic tibial anteroposterior (AP) axis connecting the centre of the posterior cruciate ligament attachment and the medial border of the patellar tendon attachment [2]. Soft‐tissue balancing was performed to achieve varus and valgus stability during extension and flexion. The iAssist system (Zimmer), a handheld accelerometer‐based navigation system, was used to aid the surgeon in achieving the desired alignment settings.

### Image acquisition and radiological analysis

Whole‐leg AP standing radiographs and CT images were obtained during preoperative planning. Additionally, postoperative whole‐leg standing radiographs, AP radiographs of the knee in the supine position and CT images were routinely acquired two weeks after the surgery. Preoperative and postoperative whole‐leg radiographs, standard orthoroentgenograms with three shots at the hip, knee and ankle joint levels [37], were taken with the patella facing anteriorly and the feet positioned anteriorly and shoulder‐width apart [18]. Radiographs were assessed immediately after being taken, and if they were found to be of insufficient quality, they were retaken. Whole‐leg standing radiographs were evaluated using OP‐A software (Fujifilm Corporation). The hip‐knee‐ankle (HKA) angle was measured preoperatively and postoperatively. The HKA angle was measured between the mechanical axes of the femur and tibia, with 0° defined as neutral and a positive value indicating varus alignment [14, 17, 31]. CT images were acquired at 1.25 mm intervals, including the hip and ankle joints (Aquilion ONE; Canon Medical Systems Inc.), and were acquired in the Digital Imaging and Communications in Medicine (DICOM) format. The DICOM data set was imported into ZedKnee software for preoperative and postoperative images. ZedKnee software has been validated as a CT‐based three‐dimensional (3D) preoperative planning and postoperative evaluation software programme for TKA [21, 47].

**Figure 1:**
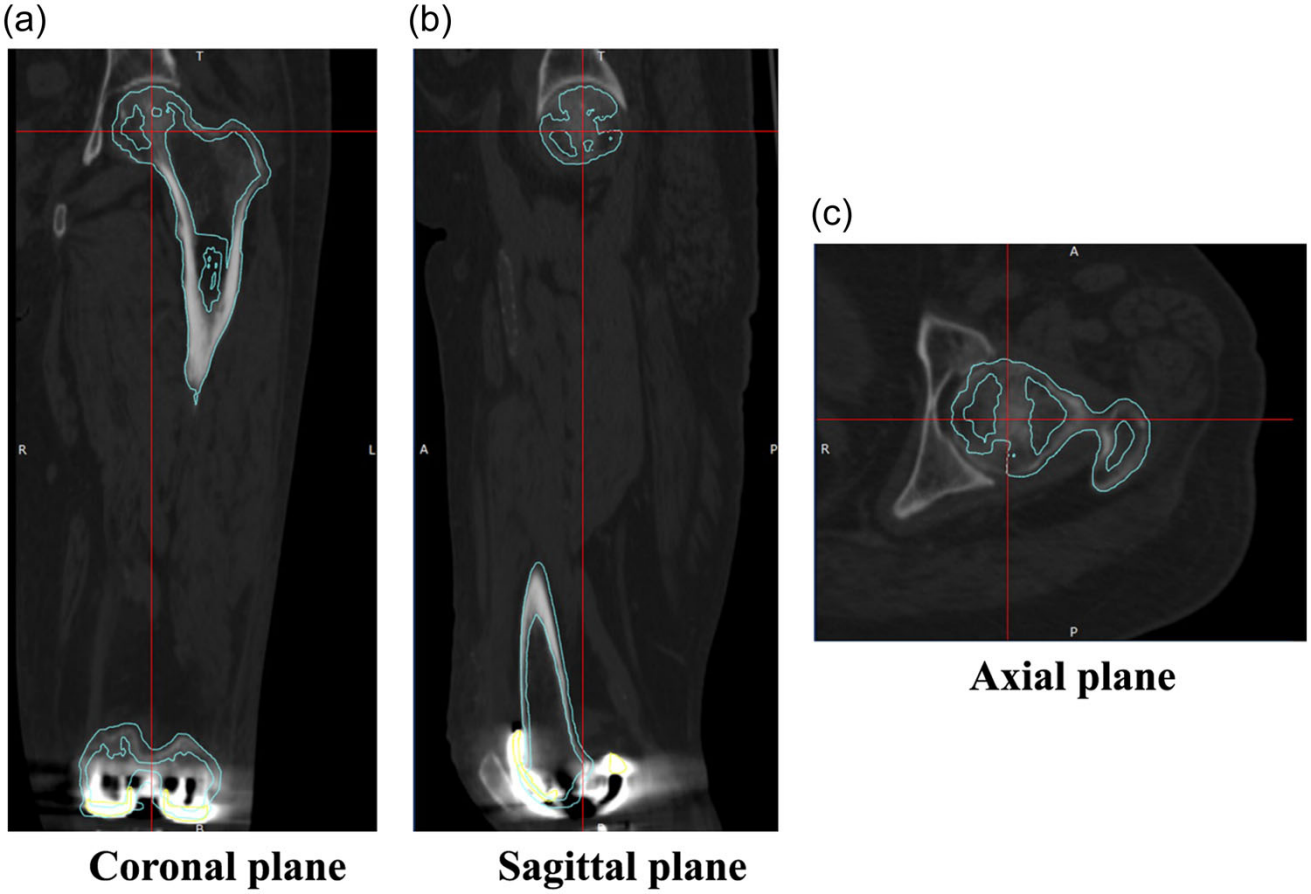
The preoperative and postoperative CT images were fused by matching the bone surfaces. Light blue lines indicate bone surfaces on preoperative CT images, while red lines indicate the reference line for each plane (a: coronal, b: sagittal and c: axial). CT, computed tomography.

The software defined the reference points on the CT images to ensure that the preoperative and postoperative 3D coordinate systems of the femur and tibia overlapped. The following points were registered in the femur: the centre of the femoral head, medial epicondylar sulcus and tip of the lateral epicondyle. The SEA was used for rotational assessment. The functional axis of the femur was defined as the line connecting the centre of the femoral head to the midpoint of the SEA. In the tibia, the following points were registered: the centre of the tibial anterior cruciate ligament (ACL) attachment, and the medial and lateral malleoli of the ankle joint. ‘Akagi's line’ was used for rotational assessment. The functional axis of the tibia was defined as the line connecting the centre of the tibial ACL attachment to the midpoint of the medial and lateral malleoli of the ankle joint. The preoperative and postoperative CT images were automatically fused by matching the bone surfaces, and the preoperative reference points and axis of rotation were projected onto the postoperative CT (Figure 1). This allowed for the evaluation of the position and rotation of the component using a common reference point before and after surgery. The alignment of the components in the coronal (component varus: +) and sagittal planes (component flexion: +) was measured as the angle between the component and the mechanical axis in both planes. The alignment in the axial plane was measured as the rotation of the femoral component from the SEA (internal rotation: +) and the rotation of the tibial component from Akagi's line (internal rotation: +). Consistent with previous reports, a malalignment exceeding 3° in either the coronal, sagittal or axial planes was defined as an outlier [20, 43].

### Evaluation of rotational alignment

**Figure 2:**
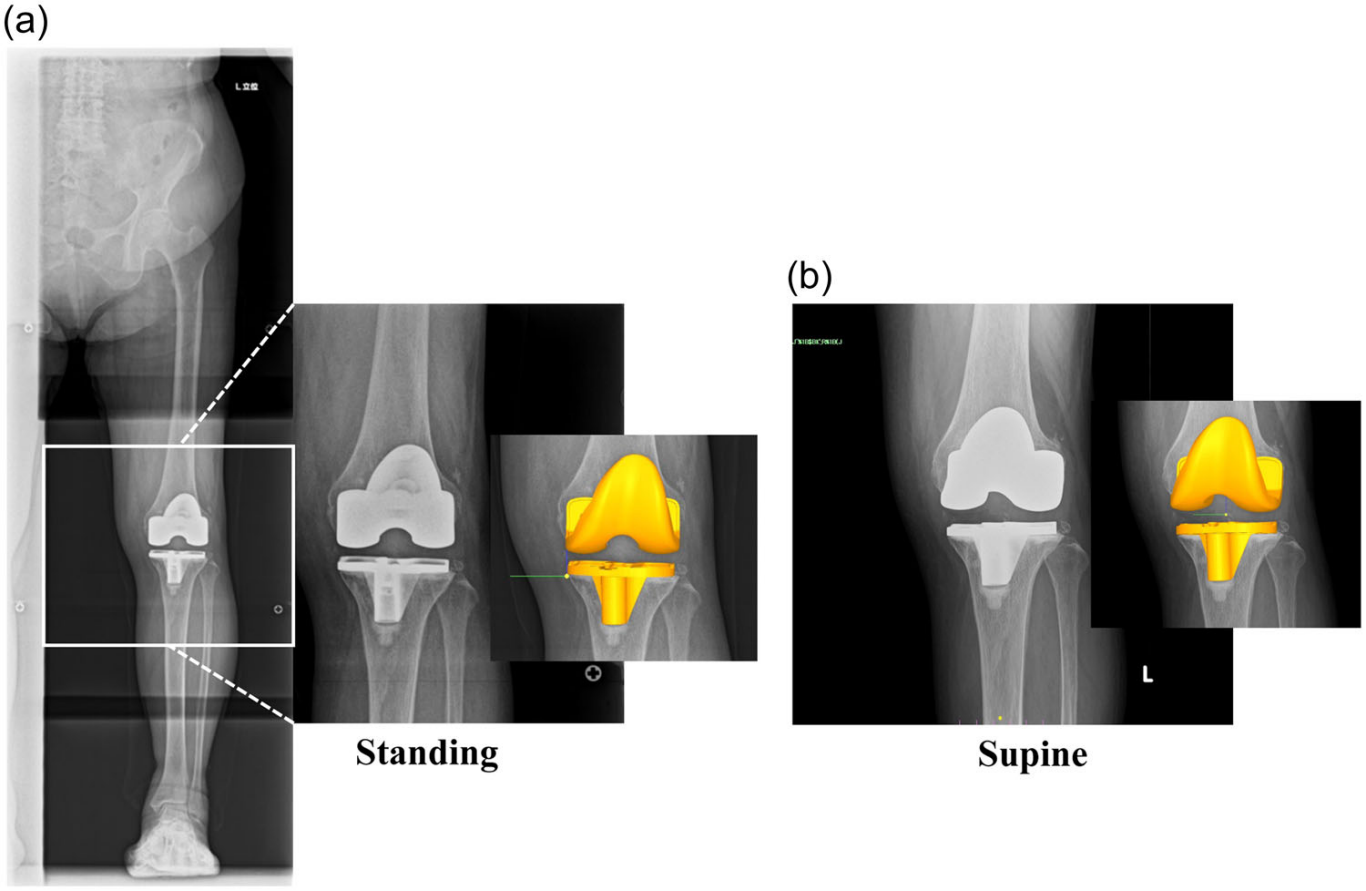
The rotational alignment between components in the standing (a) and supine (b) positions was analysed using the 3D‐to‐2D model image registration technique. The 3D CAD model of the femoral and tibial components was superimposed onto the 2D radiograph. 2D, two‐dimensional; 3D, three‐dimensional; CAD, computer‐aided design.

**Figure 3:**
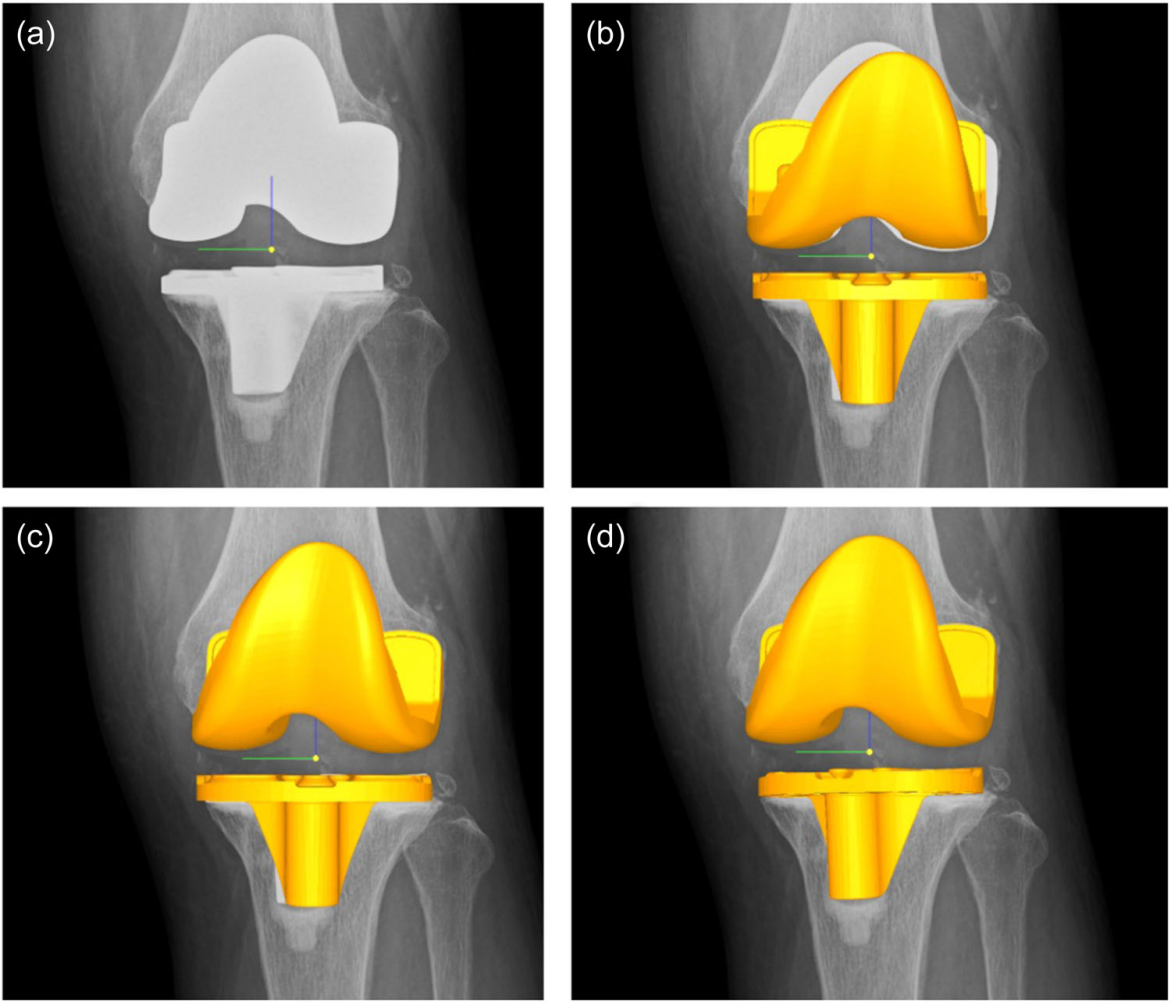
Workflow of the 3D‐to‐2D model image registration technique: (a) A knee radiograph is uploaded. (b) A 3D CAD model of the femoral and tibial components is uploaded. (c) The 3D CAD model is translated and rotated to match the silhouette of the femoral components in the radiographic image. In this case, the femoral component is rotated 2.3° in the coronal plane, −6.2° in the sagittal plane and 10.7° in the axial plane. (d) The same process is repeated with the tibial component. In this case, the tibial component was rotated −0.5° in the coronal plane, −2.3° in the sagittal plane and 23.4° in the axial plane. 2D, two‐dimensional; 3D, three‐dimensional; CAD, computer‐aided design.

The study analysed the rotational alignment between components in the standing and supine positions using a 3D‐to‐two‐dimensional (2D) model image registration technique [7, 8, 22, 25]. The DICOM datasets of the postoperative whole‐leg standing and supine knee radiographs were imported into ZedView software (Lexi Co., Ltd.) for kinetic analysis. ZedView software (dynamic analysis mode) is a validated kinetic analysis software programme for TKA that can accurately analyse length errors within 0.3 mm and angular errors within 0.25° [29, 38]. The projected 3D computer‐aided design (CAD) model of the femoral and tibial components was superimposed onto the 2D radiographic images (Figure 2). The 3D CAD model was translated and rotated to match the silhouette of the actual components of the radiographic image. Using the 3D‐to‐2D model image registration technique, the study evaluated the axial rotation of the tibiofemoral implant in the postoperative whole‐leg standing and supine knee radiographs (Figure 3). The reference axis used to assess axial rotation was established as previously described [22]. It was measured between the cylindrical axis of the posterior condyle of the femoral component and the vertical axis of an ellipse approximating the tibial baseplate. A rotational mismatch of >5° between the components was considered an outlier, in accordance with the previous report [10, 36]. A subgroup analysis was conducted to investigate the characteristics of patients whose rotational mismatch varied significantly between supine and standing positions, which represents the intraoperative component placement and functional position. The ‘underestimated group’ was defined as patients who did not have a rotational mismatch between the components in the supine position but did have a mismatch in the standing position. The ‘overestimated group’ was defined as patients who had a rotational mismatch in the supine position but not in the standing position. The control group included patients whose evaluation did not change between the supine and standing positions. Radiological parameters were compared between the underestimated and control groups, and between the overestimated and control groups.

### Data analysis

Continuous variables are presented as mean ± standard deviation. A paired *t*‐test was used to compare rotational alignment in the standing and supine positions. For comparisons of radiological parameters in the subgroup analysis, either the *t* test or Wilcoxon signed‐rank test was used as appropriate, based on the Shapiro–Wilk test result. Statistical significance was set at a *p* value < 0.05. Multivariate logistic regression analysis using a stepwise variable entry method was performed to identify the factors associated with the postoperative KSS 2011. Radiological parameters (preoperative HKA angle, alignments of femoral and tibial components in the coronal, sagittal and axial planes and rotational mismatch between components) and demographic parameters (sex and BMI) were used in the multivariate logistic regression model. Multivariate analysis was performed with continuous variables and categorical variables (whether each parameter was an outlier). A preoperative HKA angle >20° [24, 31] and BMI > 25 kg/m^2^ [45] were defined as outliers based on previous studies. For each identified factor, subgroup analyses were performed to compare KSS total scores and subscales. Differences were evaluated if they exceeded the minimal clinically important difference (MCID) values reported in previous studies: 1.9 for symptoms, 2.2 for satisfaction, 4.1 for functional activities and 10.0 for the total score [28, 34]. Statistical analyses were performed using JMP statistical analysis software (version 17.0; SAS Institute). To assess intra‐observer and inter‐observer reproducibilities, measurements were repeated twice by one examiner (YK) and once by another examiner (SK) in the study group. The intra‐ and inter‐class correlation coefficients were good (0.86 to 0.91 and 0.80 to 0.88, respectively) for all measurements (Table A1). Post hoc power analysis was conducted using G*Power version 3.1 (Heinrich‐Heine‐Universität). With a total sample size of 71 and a type‐I error (*α*) of 0.05, the study was expected to achieve power (1 − *β*) of 0.95, 0.99 and 0.99 for detecting effect sizes of 0.4, 0.5 and 0.6, respectively.

## RESULTS

**Figure 4:**
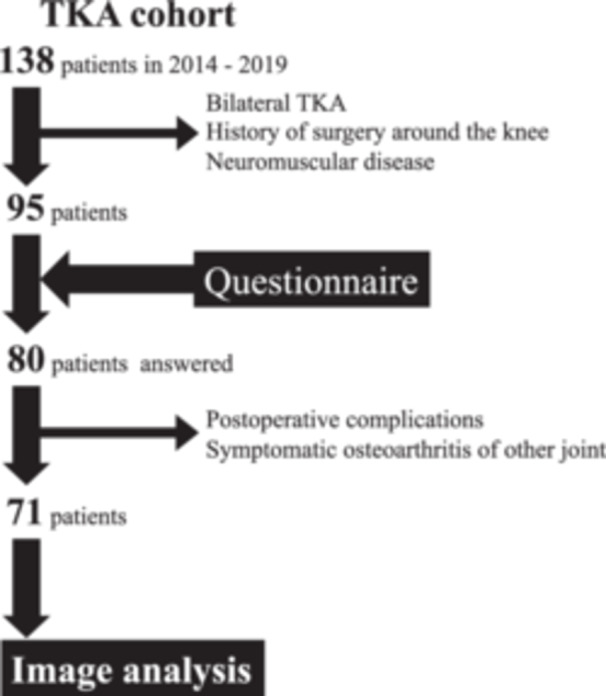
STROBE diagram illustrating the inclusion process. STROBE, Strengthening the Reporting of Observational Studies in Epidemiology. TKA, total knee arthroplasty.

In total, 138 consecutive patients (175 knees) fulfilled the inclusion criteria. After excluding 37 patients (74 knees) who underwent bilateral TKA, 4 patients (4 knees) with a history of high tibial osteotomy and 2 patients (2 knees) with a history of neuromuscular disease, questionnaires were sent to 95 patients (95 knees) who had a minimum follow‐up of two years. Out of the 95 patients, 80 (84%) completed and returned the questionnaire with written informed consent. After reviewing the medical records of these patients, the following were excluded: two patients who had postoperative complications (infection and periprosthetic fracture), two patients who had medical complications (e.g., stroke) and a significantly decreased level of activities of daily living and five patients who had symptomatic contralateral knee OA or ipsilateral hip OA. After the eligibility assessment, 71 patients (71 knees) were enroled in this study (Figure 4). All patients were Japanese. Patient demographics and preoperative and postoperative radiological data are presented in Table 1. Knee joint range of motion improved from −9.4 ± 7.6° preoperatively to −1.1 ± 2.4° postoperatively (*p* < 0.0001) in extension and from 121.1 ± 12.5° preoperatively to 129.6 ± 9.2° postoperatively (*p* < 0.0001) in flexion.

**Table 1:** Patient demographic and radiographic data.

Parameters	*n* = 71
Age (year)	74.7 ± 7.1
Sex (*n*)	Male 10, Female 61
BMI (kg/m^2^)	27.4 ± 4.4
Preoperative HKA angle (°)	9.6 ± 5.1
Coronal parameters (varus +, valgus −)	
Femoral component alignment (°)	0.4 ± 2.0
Outlier of femoral component (>3° or −3°>)	10 (14%)
Tibial component alignment (°)	0.1 ± 1.6
Outlier of tibial component (>3° or −3°>)	3 (4%)
Sagittal parameters (flexion +, extension −)	
Femoral component alignment (°)	−0.7 ± 2.0
Outlier of femoral component (>3° or −3°>)	11 (15%)
Tibial component alignment (°)	2.8 ± 2.4
Outlier of tibial component (>3° or −3°>)	13 (18%)
Axial parameters (IR+, ER−)	
Femoral component alignment (°)	−2.8 ± 3.2
Outlier of femoral component (>3° or ‐3°>)	34 (48%)
Tibial component alignment (°)	0.5 ± 6.0
Outlier of tibial component (>3° or −3°>)	47 (66%)

*Note*: Values are given as the mean and standard deviation.

Abbreviations: BMI, body mass index; ER, external rotation; HKA, hip‐knee‐ankle; IR, internal rotation.

The rotational mismatch between the components in the supine and standing positions was analysed using the 3D‐to‐2D model image registration technique. The results are displayed in Table 2. An analysis of the entire cohort showed no significant difference in the rotational mismatch of components between the supine (4.7 ± 3.1°) and standing (4.7 ± 3.7°) positions (*p* = 0.9315). In the subgroups, 44 patients (62.0%) showed no change in the rotational mismatch between the supine (4.8 ± 3.5°) and standing positions (4.9 ± 4.4°, *p* = 0.7324). Eleven patients (15.5%) belonged to the underestimated group, with a greater mismatch in the standing position (6.6 ± 1.3°) than in the supine position (2.2 ± 1.6°) (*p* < 0.0001). Conversely, 16 patients (22.5%) belonged to the overestimated group, with a smaller rotational mismatch in the standing position (3.1 ± 1.5°) than in the supine position (6.2 ± 1.3°) (*p* < 0.0001). Radiological parameters were compared between the underestimated or overestimated groups and the control group (Tables 3a and 3b). In the underestimated group, the preoperative HKA angle was greater than that in the control group (*p* = 0.0294), and both the femoral (*p* = 0.0379) and tibial components (*p* = 0.0147) were placed in external rotation. Tibial components (*p* = 0.0449) were placed in external rotation in the overestimated group.

**Table 2:** Rotational mismatch between components in the supine and standing positions.

	Supine	Standing	*p* Value
Rotational mismatch	4.7 ± 3.1	4.7 ± 3.7	0.9315
Outlier of rotational mismatch (>5°)	34 (48%)	29 (41%)	0.0566

*Note*: Values are given as the mean and standard deviation.

Abbreviation: SD, standard deviation.

**Table 3a:** Patient radiographic data in the underestimated and normal groups.

Parameters	Underestimated (*n* = 11)	Control (*n* = 44)	*p* Value
Preoperative HKA angle (°)	11.7 ± 6.2	8.5 ± 4.6	**0.0294**
Coronal parameters			
Femoral component alignment (°)	0.4 ± 1.7	0.76 ± 2.4	0.7917
Tibial component alignment (°)	0.2 ± 1.4	0.1 ± 1.7	0.5555
Sagittal parameters			
Femoral component alignment (°)	−1.8 ± 1.7	−0.5 ± 2.0	0.0675
Tibial component alignment (°)	2.7 ± 2.5	3.0 ± 2.8	0.4362
Axial parameters			
Femoral component alignment (°)	−4.4 ± 2.4	−2.2 ± 3.3	**0.0379**
Tibial component alignment (°)	−3.6 ± 5.9	2.2 ± 6.1	**0.0147**
Component mismatch			
Rotational mismatch (supine, °)	2.2 ± 1.6	4.8 ± 3.5	**0.0294**
Rotational mismatch (standing, °)	6.6 ± 1.3	4.9 ± 4.4	**0.0343**

*Note*: Values are given as the mean and standard deviation. The bold values indicates statistically significant at *p* < 0.05.

Abbreviation: HKA, hip‐knee‐ankle.

**Table 3b:** Patient radiographic data in the overestimated and normal groups.

Parameters	Overestimated (*n* = 16)	Control (*n* = 44)	*p* Value
Preoperative HKA angle (°)	11.4 ± 5.1	8.5 ± 4.6	0.0611
Coronal parameters			
Femoral component alignment (°)	0.1 ± 2.2	0.4 ± 1.7	0.6320
Tibial component alignment (°)	0.3 ± 1.4	0.1 ± 1.7	0.8540
Sagittal parameters			
Femoral component alignment (°)	−0.5 ± 2.3	−0.5 ± 2.0	0.8059
Tibial component alignment (°)	3.0 ± 1.8	2.7 ± 2.5	0.5473
Axial parameters			
Femoral component alignment (°)	−3.3 ± 3.2	−2.2 ± 3.3	0.2248
Tibial component alignment (°)	−1.2 ± 4.0	2.2 ± 6.1	**0.0449**
Component mismatch			
Rotational mismatch (supine, °)	6.2 ± 1.3	4.8 ± 3.5	**0.0324**
Rotational mismatch (standing, °)	3.1 ± 1.5	4.9 ± 4.4	0.3667

*Note*: Values are given as the mean and standard deviation. The bold values indicates statistically significant at *p* < 0.05.

Abbreviation: HKA, hip‐knee‐ankle.

Tables 4a and 4b present the multivariable analysis results for postoperative KSS 2011. The study found that the rotational mismatch between components in the standing position was a significant risk factor for unfavourable postoperative KSS 2011. Specifically, patients with a rotational mismatch in the standing position had significantly lower scores on the total score (*p* = 0.0215), symptom subscale (*p* = 0.0255) and functional activity subscale (*p* = 0.0416). Importantly, each of these differences exceeded the MCID (Table 5).

**Table 4a:** Multivariate analysis of factors associated with the postoperative KSS 2011 (performed with continuous variables).

Parameters	*β* value (95% CI)	*p* Value
Rotational mismatch in standing (°)	**−3.06 (−4.51 to −0.95)**	**0.0032**
Tibial component alignment in coronal plane (°)	1.82 (−0.35 to 7.83)	0.0726
BMI (kg/m^2^)	1.61 (−0.29 to 2.68)	0.1117

*Note*: Multivariate logistic regression analysis was performed using a stepwise variable entry method. Radiological parameters (preoperative HKA angle, alignments of femoral and tibial components in the coronal, sagittal and axial planes, and rotational mismatch between components) and demographic parameters (sex, BMI) were used in the multivariate logistic regression model.

*β* is the standard regression coefficient. The bold values indicates statistically significant at *p* < 0.05.

Abbreviations: BMI, body mass index; CI, confidence interval; KSS, Knee Society Score.

**Table 4b:** Multivariate analysis of factors associated with the postoperative KSS 2011 (performed with categorical variables).

Parameters	*β* value (95% CI)	*p* Value
Outlier of mismatch in standing (>5°)	**−2.49 (−15.39 to −1.70)**	**0.0152**
Outlier of coronal femoral component (>3° or −3°>)	1.70 (−1.45 to 17.83)	0.0948
Outlier of axial femoral component (>3° or −3°>)	−1.83 (−12.80 to 0.54)	0.0711
Outlier of BMI (>25 kg/m^2^)	1.36 (−2.24 to 11.75)	0.1791

*Note*: Multivariate logistic regression analysis was performed using a stepwise variable entry method. Radiological and demographic parameters (preoperative HKA angle, alignments of femoral and tibial components in the coronal, sagittal and axial planes, and rotational mismatch between components, sex and BMI; whether the parameter was an outlier or not) were used in the multivariate logistic regression model.

*β* is the standard regression coefficient. The bold values indicates statistically significant at *p* < 0.05.

Abbreviations: BMI, body mass index; CI, confidence interval; KSS, Knee Society Score.

**Table 5:** Comparison of the postoperative KSS 2011 with (>5°) and without (<5°) rotational mismatch between components.

	Mismatch in standing (>5°) (*n* = 29)	Mismatch in standing (<5°) (*n* = 42)	*p* Value
Total score of KSS 2011 (3–180)	113.9 ± 33.1	130.1 ± 25.0	**0.0215**
Subscales			
Symptom (0–5)	19.0 ± 5.5	21.6 ± 4.0	**0.0255**
Satisfaction (0–40)	25.2 ± 7.6	28.7 ± 7.2	0.0719
Expectation (3–15)	9.9 ± 3.7	10.4 ± 3.3	0.8070
Functional activity (0–100)	59.8 ± 23.3	69.5 ± 16.0	**0.0416**

*Note*: Values are given as the mean and standard deviation. The bold values indicates statistically significant at *p* < 0.05.

Abbreviation: KSS, Knee Society Score.

## DISCUSSION

The most important finding of this study was that a rotational mismatch between components in the standing position was a potential risk factor for unfavourable postoperative PROMs. However, the rotational mismatch between components in the standing position has not been extensively studied due to the difficulty in accurately quantifying rotational alignment from standard radiographs and CT images. Therefore, the present study utilized a 3D‐to‐2D model image registration technique to measure rotational alignment from radiographs taken in both the supine and standing positions. This technique has been previously used to analyse hip and knee joint kinetics using fluoroscopic images of daily activities, such as walking and squatting, demonstrating its high accuracy [9, 25, 46]. It has recently been applied to assess changes in axial alignment between the supine and standing positions in patients after total hip arthroplasty [42] or the intraoperative acetabular component in total hip arthroplasty [15]. This is the first study that used this technique to evaluate changes in knee rotational alignment from supine to standing. The recently reported standing CT may also be another method for assessing rotational mismatch between components in the standing position [11, 32]. Rotational alignment is affected by quadriceps contraction [11, 26] and soft‐tissue laxity around the knee joint [35] during a position change from supine to standing.

There was no significant difference in the rotation mismatch between the supine and standing positions in the entire cohort. In the overestimated group, the rotational mismatch was smaller in the standing than in the supine position. These results demonstrate the concept of component design that compensates for the self‐aligned rotation mismatch [10, 41]. However, in some cases (underestimated group), the rotational mismatch was greater in the standing than in the supine position, even though the rotational mismatch was small in the supine position. In the underestimated group, the preoperative varus deformity was significantly greater, and both the femoral and tibial components exhibited external rotation. In contrast, in the control group, the femoral components displayed external rotation, while the tibial components showed internal rotation. This can be explained by the effects of soft‐tissue laxity [6, 13, 23, 40]. All the patients in the cohort were treated using the measured resection technique with mechanical alignment. The medial release was minimized; however, in cases of severe preoperative varus deformity, soft‐tissue release was sometimes increased, creating an extension gap [13, 40]. Excessive soft‐tissue release is also necessary to correct malrotation of the femoral component and create a flexion gap. However, it is important to avoid excessive medial release, as it may result in hypermobility under dynamic conditions. Furthermore, in patients with severe varus deformity, the flexion gap tends to be tight, and the femoral component may have been aligned in external rotation to create a flexion gap, even when the surgery was performed using the measured resection technique. Moreover, it has been reported that external rotation of the tibial component, as opposed to internal rotation, reduces both MCL tension [6] and quadriceps force by decreasing the *Q*‐angle [23]. These factors may also contribute to the rotational mismatch between components when transitioning from the supine to standing positions, attributable to variations in soft‐tissue laxity.

To the best of our knowledge, this study is the first to investigate the relationship between rotational malalignment in the standing position and postoperative PROMs. Symptoms associated with rotational misalignment include patellar tracking, joint stability and anterior knee pain, all of which occur in the weight‐bearing position rather than in the supine position [1, 3]. This study is particularly relevant as it focuses on evaluating images under weight‐bearing conditions, which may provide an explanation for these dynamic symptoms. The postoperative KSS 2011 includes several questions that specifically inquire about the condition of the knee in the standing position, making it crucial to assess rotational alignment for functional evaluation. The rotational mismatch between the components in the standing position is a significant risk factor for unfavourable PROMs. Treatment of the underestimated group is clinically important because the surgical procedure and intraoperative assessment of component alignment are performed in the supine position. In this group, the rotational mismatch is small in the supine position but large in the standing position, which is the functional position. Therefore, as mentioned earlier, we recommend minimizing medial release in cases with severe preoperative varus deformity, using surgical navigation systems or robotic‐arm‐assisted systems [4, 16, 19, 36], and relying on various perioperative landmarks [17] to avoid rotational malalignments of the components. Results of previous biomechanical studies are also consistent with the current findings [1, 10]. A rotation mismatch of >5° between the components is reported to cause excessive external rotation of the femur in mid‐flexion during knee flexion motion [1, 10]. Hirschmann et al. performed upright weight‐bearing CT scans of the knee during flexion. They reported that as the knee flexes and the femur externally rotates, the patellofemoral external rotation decreases and the patellofemoral distance also decreases [11]. Therefore, a rotational mismatch of >5° could increase femoral external rotation and result in anterior knee pain associated with the patellofemoral joint [1, 10, 11]. The present study had several limitations. This study included only one implant design, and all procedures involved posterior‐stabilized TKA. Therefore, the results of this study may not necessarily apply to other implant designs or cruciate‐retaining TKA. All the surgeries followed a standardized measured resection technique, which used anatomical landmarks, such as the SEA and Akagi's line, to guide component rotational alignment. In contrast to this surgical technique, the ‘Range of motion (ROM) technique’ has been reported to help reduce the postoperative component rotational mismatch [5, 27]. In the ROM technique, the knee is moved through a full range of flexion and extension, enabling the tibial component trial to align itself optimally with the femoral component [5, 27]. Using this technique can prevent the rotational mismatch between components, but there is controversy over which technique is preferable. Additionally, our study included a limited number of patients [12]. Although significant *p* values may imply an adequate sample size for detecting effects, it is essential to conduct further analysis with larger cohorts to generalize the study findings. The study excluded knees with valgus OA and the preoperative PROMs were not evaluated. Patients exhibiting poor preoperative PROMs, particularly those with pronounced preoperative deformities, might also demonstrate suboptimal postoperative PROMs. Finally, the postoperative measurement was only conducted once, two weeks after TKA. This is relatively early compared to the questionnaires and clinical evaluations. It is possible that soft tissue had healed by then, potentially leading to increased resistance against rotational mismatch. Therefore, further longitudinal studies are needed to observe rotational mismatch.

## CONCLUSIONS

In 62.0% of patients, the rotational mismatch between components remained unchanged between the supine and standing positions. However, in cases with severe preoperative varus deformity or malrotation of the femoral and tibial components, a large rotational mismatch between components can occur when in the standing position, even if it was evaluated as small in the supine position. This rotational mismatch between components in the standing position has the potential to be a risk factor for unfavourable postoperative PROMs. Therefore, caution should be exercised to avoid placing the component in malrotation when dealing with severe preoperative varus deformity, to prevent rotational mismatch when in the standing position.

## AUTHOR CONTRIBUTIONS

Substantial contributions to research design, or the acquisition, analysis or interpretation of data: Yasuhiko Kokubu, Shinya Kawahara, Hideki Mizu‐Uchi, Satoshi Hamai, Yukio Akasaki, Taishi Sato, Shojiro Ishibashi, Toshiki Konishi and Yasuharu Nakashima. Drafting the paper or revising it critically: Yasuhiko Kokubu and Shinya Kawahara. Approval of the submitted and final versions: Yasuhiko Kokubu, Shinya Kawahara, Hideki Mizu‐Uchi, Satoshi Hamai, Yukio Akasaki, Taishi Sato, Shojiro Ishibashi, Toshiki Konishi and Yasuharu Nakashima.

## CONFLICT OF INTEREST STATEMENT

The authors declare no conflict of interest.

## ETHICS STATEMENT

The Institutional Review Board approved the current study (No. 2020‐204). All patients included in this study provided informed consent.

## Data Availability

The data sets used and analyzed during the current study are available from the corresponding author on reasonable request.

## APPENDIX A

**Table A1:** Intra‐ and inter‐class correlation coefficients of radiographic data.

Parameters	Intra‐class correlation (95% CI)	Inter‐class correlation (95% CI)
Preoperative HKA angle	0.90 (0.78–0.96)	0.88 (0.86–0.90)
Coronal parameters		
Femoral component alignment	0.91 (0.84–0.94)	0.81 (0.64–0.87)
Tibial component alignment	0.88 (0.72–0.96)	0.88 (0.82–0.92)
Sagittal parameters		
Femoral component alignment	0.91 (0.74–0.97)	0.84 (0.77–0.89)
Tibial component alignment	0.86 (0.79–0.91)	0.83 (0.76–0.88)
Axial parameters		
Femoral component alignment	0.88 (0.74–0.94)	0.80 (0.67–0.94)
Tibial component alignment	0.86 (0.76–0.94)	0.83 (0.72–0.89)
Component mismatch		
Rotational mismatch (supine)	0.90 (0.82–0.94)	0.84 (0.77–0.89)
Rotational mismatch (standing)	0.86 (0.79–0.92)	0.86 (0.79–0.91)

*Note*: Values are given as the mean and standard deviation.

Abbreviations: CI, confidence interval; HKA, hip‐knee‐ankle.
